# Serum bilirubin level is associated with exercise capacity and quality of life in chronic obstructive pulmonary disease

**DOI:** 10.1186/s12931-019-1241-5

**Published:** 2019-12-09

**Authors:** Ah Young Leem, Young Sam Kim, Ji-Hyun Lee, Tae-Hyung Kim, Ha Yan Kim, Yeon Mok Oh, Sang Do Lee, Ji Ye Jung

**Affiliations:** 1 Division of Pulmonology, Department of Internal Medicine, Institute of Chest Disease, Yonsei University College of Medicine, 50-1 Yonsei-ro, Seodaemun-gu, Seoul, 03722 Republic of Korea; 2 Department of Internal Medicine, CHA Bundang Medical Center, CHA University, Seongnam, Republic of Korea; 3 Division of Pulmonary and Critical Care Medicine, Hanyang University Guri Hospital, Hanyang University College of Medicine, Guri, Republic of Korea; 4 Biostatistics Collaboration Unit, Yonsei University College of Medicine, Seoul, Republic of Korea; 5 Department of Pulmonary and Critical Care Medicine and Clinical Research Center for Chronic Obstructive Airway Diseases, Asan Medical Center, University of Ulsan College of Medicine, Seoul, Republic of Korea

**Keywords:** Chronic obstructive pulmonary disease, Exercise, Quality of life, Bilirubin, Exacerbation

## Abstract

**Background:**

Bilirubin has antioxidant properties against chronic respiratory diseases. However, previous studies are limited by acquisition of serum bilirubin level at one time point and its analysis with clinical parameters. We evaluated the association of serum bilirubin levels with various clinical outcomes of chronic obstructive pulmonary disease (COPD) in Korean Obstructive Lung Disease (KOLD) cohort.

**Methods:**

We included 535 patients with COPD from the KOLD cohort. Serum bilirubin levels and various clinical parameters, such as lung function, 6-min walking (6 MW) distance, quality of life (QoL), and exacerbation, were evaluated annually; their association was analyzed using generalized estimating equations and the linear mixed model.

**Results:**

Among 535 patients, 345 (64.5%) and 190 (35.5%) were categorized into Global Initiative for Chronic Obstructive Lung Disease (GOLD) I-II and GOLD III-IV groups, respectively. 6 MW distance was positively associated with serum bilirubin levels, especially in the GOLD I-II group (estimated mean = 41.5). Among QoL indexes, the COPD assessment test score was negatively associated with serum bilirubin levels only in the GOLD I-II group (estimated mean = − 2.8). Higher serum bilirubin levels were independently associated with a higher number of acute exacerbation in the GOLD III-IV group (estimated mean = 0.45, *P* = 0.001). Multivariate analysis revealed that lung function and mortality were not associated with serum bilirubin levels.

**Conclusions:**

Higher serum bilirubin levels were associated with a longer 6 MW distance and better QoL, especially in the GOLD I-II group, whereas they were related to a higher risk of acute exacerbation, especially in the GOLD III-IV group. Bilirubin levels may represent various conditions in COPD.

## Background

Chronic obstructive pulmonary disease (COPD) is characterized by persistent respiratory symptoms and airflow limitation caused by significant exposure to noxious particles or gases [1]. Oxidative stress is an important mechanism in the development, progression, and exacerbation of COPD. Biomarkers of oxidative stress are elevated in the exhaled breath, sputum, and blood of patients with COPD [2].

Bilirubin is known as a potential antioxidant and possesses anti-inflammatory properties [3]. Elevated bilirubin levels have a protective effect in cardiovascular disease and cardiovascular disease–related diseases [4]. Several reports demonstrate the relationship between bilirubin level and respiratory diseases. In the United Kingdom, higher level of bilirubin was associated with a lower risk of COPD, lung cancer, and all-cause mortality [5]. In the Swiss general population, serum bilirubin was positively associated with lung function [6]. In COPD, bilirubin level was inversely related to COPD disease severity and progression, and a higher bilirubin level was associated with a lower risk for acute COPD exacerbation, suggesting that bilirubin can be a biomarker of COPD exacerbation [7, 8].

However, previous studies have a potential limitation with respect to the acquisition of serum bilirubin level at one time point and analyzing it with serial clinical parameters. Brown et al. analyzed the serum bilirubin level through repeated measurements, but the interval of measurement and total duration of the study were too short to assess the relationship of serum bilirubin level with various clinical outcomes in COPD [8]. For comparison with the results of previous conflict results, we aimed to evaluate the association of both baseline and repeatedly measured serum bilirubin levels with various clinical outcomes of COPD in Korea, including lung function, quality of life (QoL), exercise capacity, exacerbation, and mortality.

## Methods

### Study subjects

The study population initially consisted of 547 patients from the KOLD cohort, in which patients with COPD or asthma had been recruited from the pulmonary clinics of 17 hospitals in South Korea between June 2005 and December 2011 and are under continuous follow-up. The inclusion criteria were a postbronchodilator ratio of forced expiratory volume in 1 s to forced vital capacity (FEV_1_/FVC) < 0.7, age > 40 years, smoking history ≥10 pack-years, and no or minimal abnormality on chest radiography. Twelve patients were excluded (one had a high bilirubin level, nine were hepatitis B virus carriers, and two patients had moderate-to-severe liver diseases). The severity of disease was based on the FEV_1_% predicted in accordance with previous Global Initiative for Chronic Obstructive Lung Disease (GOLD) criteria [1].

### Bilirubin measurement

Venous blood samples were collected from the patients at baseline and annually, unless they refused for any reason. A high bilirubin level was defined as a bilirubin concentration > 2.34 mg/dL for men and > 1.75 mg/dL for women [9]. These limits represent concentrations 1 standard deviation (SD) above the mean serum total bilirubin related to the most common variant of Gilbert syndrome, a benign hereditary cause of indirect hyperbilirubinemia [5].

### Clinical assessments

Clinical assessments include pulmonary function test results, 6-min walking (6 MW) distance, QoL measured by COPD Assessment Test (CAT) and St. George’s Respiratory Questionnaire (SGRQ), exacerbations, and mortality. These assessments were performed annually on the same day as blood collection. The 6 MW test was conducted on a 20-m walking course [10]. Exacerbation was defined as an unscheduled hospital visit or hospitalization due to aggravation of one of three respiratory symptoms, including cough, sputum, and dyspnea for 2 days or more.

### Statistical analysis

We assessed the association between serum bilirubin levels and clinical outcomes. First, to assess the baseline bilirubin level that effects changes in lung function, 6 MW distance, CAT and SGRQ scores; and exacerbation over time, we applied the linear mixed model and generalized estimating equations (GEE) approach to the dichotomous and continuous outcomes, respectively for regression analysis. Second, the marginal GEE approach was implemented to account for the clustered measured clinical outcomes and serum bilirubin levels in the same patient for regression analysis. Age, sex, body mass index (BMI), smoking status, and baseline FEV_1_ were included in the model. We also examined the serum bilirubin levels that affect mortality through the Cox regression model using time-dependent repeated measurements. Statistical analysis was conducted with SAS version 9.4 (SAS Institute, Inc., Cary, NC, USA). We performed univariate analyses with the χ^2^ test for categorical variables and Student’s t-test for continuous variables. A two-sided *P*-value < 0.05 was considered statistically significant.

## Results

### Baseline characteristics

The serum bilirubin levels did not vary between visits over the follow-up period [see Additional file 1]. Table 1 shows the baseline characteristics of the whole study group and the GOLD I-II/III-IV groups. Among the 535 patients, 345 (64.5%) and 190 (35.5%) were categorized into GOLD I-II and GOLD III-IV groups, respectively. The mean age was 67.9 years, 97% were male, 35.8% were current smokers, the mean FEV_1_ was 1.72 L (56.8% of predicted), the mean follow-up duration was 5.4 years, and the mean bilirubin level was 0.68 ng/mL in the whole group. BMI, lung function, and proportion of current smokers were higher, and the 6 MW distance was longer in the GOLD I-II group than in the GOLD III-IV group. QoL was better in the GOLD I-II group than in the GOLD III-IV group according to CAT and SGRQ scores. The number of exacerbations and the mortality rate were higher in the GOLD III-IV group than in the GOLD I-II group. Serum bilirubin level was not different between the GOLD I-II and GOLD III-IV groups.

**Table 1:** Baseline characteristics of the KOLD study population^a^

Parameters	Whole Group (*n* = 535)	GOLD I-II (*n* = 345)	GOLD III-IV (*n* = 190)	*P*-value†
Age, yr	67.9 ± 7.9	67.8 ± 8.4	67.9 ± 6.8	0.933
Sex, male	519 (97.0)	333 (96.5)	186 (97.9)	0.438
BMI, kg/m^2^	22.9 ± 3.0	23.5 ± 2.7	21.9 ± 3.3	< 0.001
Smoking				
Current smokers	191 (35.8)	138 (40.0)	53 (28.0)	0.006
Former smokers	344 (64.2)	207 (60.0)	137 (72.0)	
Lung function				
FEV_1_, L	1.72 ± 0.59	2.02 ± 0.48	1.16 ± 0.28	< 0.001
FEV_1_, % predicted	56.8 ± 16.6	66.7 ± 10.9	38.9 ± 8.0	< 0.001
FVC, L	3.55 ± 0.79	3.78 ± 0.74	3.13 ± 0.70	< 0.001
FVC, % predicted	89.9 ± 17.1	94.6 ± 15.6	81.4 ± 16.5	< 0.001
FEV_1_/FVC, %	47.9 ± 11.2	53.4 ± 8.3	38.1 ± 8.8	< 0.001
GOLD classification				
GOLD I	41 (7.7)	41 (11.9)	0	< 0.001
GOLD II	304 (56.8)	304 (88.1)	0	
GOLD III	163 (30.5)	0	163 (85.8)	
GOLD IV	27 (5.0)	0	27 (14.2)	
Laboratory findings				
White blood cell, /mm3	7282 ± 2058	7087 ± 1772	7627 ± 2346	0.006
Red blood cell, /mm3	4691 ± 444	4646 ± 445	4754 ± 441	0.012
Hemoglobin, g/dl	14.7 ± 1.3	14.6 ± 1.3	14.8 ± 1.3	0.071
AST, IU/L	24.0 ± 10.9	24.8 ± 12.5	22.8 ± 7.6	0.057
ALT, IU/L	21.7 ± 15	22.9 ± 17.7	20.2 ± 9.6	0.07
Baseline bilirubin level, ng/mL	0.68 ± 0.29	0.67 ± 0.29	0.69 ± 0.29	0.671
6 MW distance, m	413.1 ± 88.1	422.4 ± 81.2	399.4 ± 92.3	0.004
CAT score	12.0 ± 7.8	10.5 ± 7.0	15.8 ± 8.2	< 0.0001
SGRQ score	30.3 ± 18.4	25.1 ± 16.2	39.1 ± 18.5	< 0.0001
Exacerbation, yr	0.33 ± 0.68	0.22 ± 0.40	0.53 ± 0.97	< 0.0001
Follow-up duration, yr	5.4 ± 3.7	5.2 ± 3.6	5.9 ± 3.9	0.031
Mortality	58 (10.8)	23 (6.7)	35 (18.4)	< 0.001

^a^ Data are presented as numbers (percentages) or means ± standard deviation

† *P*-value for comparison between COPD patients with GOLD I-II and those with GOLD III-IV

*BMI* body mass index, *FEV*_*1*_ forced expiratory volume in 1 s, *FVC* forced vital capacity, *GOLD* Global Initiative for Chronic Obstructive Lung Disease, *AST* aspartate aminotransferase, *ALT* alanine aminotransferase, *6 MW* 6-min walking, *CAT* COPD assessment test, *SGRQ* St. George’s Respiratory Questionnaire

### Pulmonary function

Table 2 shows the results of the regression analysis of the relationship of serum bilirubin levels and pulmonary function. Baseline serum bilirubin level was associated with FEV_1_ (estimated mean = 0.19, *P* = 0.038) and FVC (estimated mean = 0.34, *P* = 0.017) in the GOLD I-II group according to the univariate analysis. However, the multivariate analysis adjusted for age, sex, BMI, and smoking status showed no significant association between serum bilirubin levels and pulmonary function in the analysis of the baseline level or in the clustered analysis.

**Table 2:** Regression analysis for the relationship between serum bilirubin levels and pulmonary function^a^

	FEV_1_, L		FVC, L		FEV_1_/FVC, %	
	estimated mean (SE)	*P*-value	estimated mean (SE)	*P*-value	estimated mean (SE)	*P*-value
Whole group						
Univariate						
Baseline bilirubin	0.08 (0.09)	0.382	0.18 (0.12)	0.121	− 0.52 (1.84)	0.776
Clustered analysis	0.05 (0.09)	0.585	0.13 (0.11)	0.255	− 0.33 (1.75)	0.85
Multivariate^b^						
Baseline bilirubin	0.04 (0.08)	0.607	0.11 (0.11)	0.322	−0.45 (1.72)	0.792
Clustered analysis	0.02 (0.08)	0.798	0.05 (0.10)	0.662	0.08 (1.55)	0.959
GOLD I-II						
Univariate						
Baseline bilirubin	0.19 (0.09)	0.038	0.34 (0.14)	0.017	0.23 (1.76)	0.898
Clustered analysis	0.14 (0.09)	0.115	0.21 (0.14)	0.116	1.21 (1.52)	0.428
Multivariate^b^						
Baseline bilirubin	0.11 (0.08)	0.169	0.19 (0.12)	0.111	−0.08 (1.69)	0.962
Clustered analysis	0.06 (0.07)	0.428	0.07 (0.12)	0.561	0.94 (1.36)	0.489
GOLD III-IV						
Univariate						
Baseline bilirubin	−0.04 (0.09)	0.622	−0.02 (0.18)	0.925	−0.52 (2.39)	0.829
Clustered analysis	−0.11 (0.08)	0.169	−0.02 (0.15)	0.916	−2.79 (2.55)	0.273
Multivariate^b^						
Baseline bilirubin	−0.07 (0.08)	0.349	−0.06 (0.17)	0.708	−0.91 (2.31)	0.694
Clustered analysis	−0.12 (0.07)	0.103	−0.06 (0.14)	0.682	−2.59 (2.42)	0.285

^a^ ‘baseline bilirubin’ is the results of regression analysis for clinical outcomes according to the baseline bilirubin levels. ‘Clustured analysis’ is the results of regression analysis for clinical outcomes and repeated measured serum bilirubin levels

^b^ adjusted with age, sex, body mass index, smoking (current and former) *FEV*_*1*_ forced expiratory volume in 1 s, *SE* standard error, *FVC* forced vital capacity, *GOLD* Global Initiative for Chronic Obstructive Lung Disease

### 6 MW distance and QoL

Table 3 shows the results of the regression analysis for the association of serum bilirubin levels with 6 MW distance and QoL index. In the whole group, the 6 MW distance (estimated mean = 31.0, *P* = 0.005) was positively associated with serum bilirubin level in the multivariate clustered analysis. According to the subgroup analysis, this positive association was observed only in the GOLD I-II group (estimated mean = 41.5, *P* = 0.002). Among the QoL index, SGRQ score was not associated with serum bilirubin level in the analysis of baseline level or in the clustered analysis. However, the CAT score (estimated mean = − 1.9, *P* = 0.048) was negatively associated with serum bilirubin level in the multivariate clustered analysis in the whole group. A negative association between the serum bilirubin level and the CAT score (estimated mean = − 2.8, *P* = 0.017) was observed only in the GOLD I-II group when subgroup analysis was conducted.

**Table 3:** Regression analysis for the association of serum bilirubin levels with the 6-min walking distance and quality of life index^a^

	6 MW distance, m		CAT		SGRQ	
	estimated mean (SE)	*P*-value	estimated mean (SE)	*P*-value	estimated mean (SE)	*P*-value
Whole group						
Univariate						
Baseline bilirubin	30.1 (14.3)	0.036	0.3 (1.3)	0.835	2.5 (2.9)	0.388
Clustered analysis	42.3 (12.5)	0.001	−2.40 (1.1)	0.028	−0.9 (2.6)	0.735
Multivariate^b^						
Baseline bilirubin	20.5 (12.2)	0.094	0.4 (1.2)	0.746	2.9 (2.5)	0.261
Clustered analysis	31.0 (11.0)	0.005	−1.9 (0.9)	0.048	0.5 (2.2)	0.826
GOLD I-II						
Univariate						
Baseline bilirubin	38.6 (16.8)	0.023	−2.8 (1.5)	0.057	−2.2 (3.2)	0.498
Clustered analysis	52.4 (15.0)	0.001	−3.5 (1.2)	0.003	−3.1 (2.5)	0.218
Multivariate^b^						
Baseline bilirubin	27.6 (14.8)	0.062	−2.1 (1.5)	0.154	−0.6 (3.2)	0.844
Clustered analysis	41.5 (13.3)	0.002	−2.8 (1.2)	0.017	−1.3 (2.6)	0.61
GOLD III-IV						
Univariate						
Baseline bilirubin	22.9 (25.4)	0.367	3.6 (2.2)	0.105	7.5 (4.6)	0.111
Clustered analysis	27.7 (19.8)	0.162	−0.7 (1.6)	0.666	2.3 (4.1)	0.569
Multivariate^b^						
Baseline bilirubin	26.7 (20.4)	0.192	2.9 (1.9)	0.139	6.4 (4.2)	0.128
Clustered analysis	20.6 (16.8)	0.219	−0.3 (1.2)	0.818	2.1 (3.0)	0.49

^a^ ‘baseline bilirubin’ is the results of regression analysis for clinical outcomes according to the baseline bilirubin levels. ‘Clustured analysis’ is the results of regression analysis for clinical outcomes and repeated measured serum bilirubin levels

^b^ Adjusted for age, sex, body mass index, smoking (current and former), and baseline forced expiratory volume in 1 s

*6 MW* 6-min walking, *CAT* COPD assessment test, *SGRQ* St. George’s Respiratory Questionnaire, *SE* standard error, *GOLD* Global Initiative for Chronic Obstructive Lung Disease

### Exacerbation

Table 4 shows the relationship between serum bilirubin levels and the number of acute exacerbation per year. In the whole group, a higher baseline serum bilirubin level was independently associated with a higher risk of exacerbation (estimated mean = 0.62, *P* = 0.001). According to the subgroup analysis, this significant relationship was observed only in the GOLD III-IV group. Number of acute exacerbation per year was positively associated with serum bilirubin both in the analysis of baseline bilirubin level (estimated mean = 0.75, *P* = 0.005) and in the clustered analysis (estimated mean = 0.45, *P* = 0.001) in the GOLD III-IV group (Table 4).

**Table 4:** Regression analysis for the relationship between serum bilirubin levels and number of acute exacerbation per year^a^

	Whole Group		GOLD I-II		GOLD III-IV	
	Estimated mean (SE)	*P*-value	Estimated mean (SE)	*P*-value	Estimated mean (SE)	*P*-value
Univariate						
Baseline bilirubin	0.64 (0.19)	0.001	0.38 (0.23)	0.103	0.86 (0.27)	0.002
Clustered bilirubin	0.30 (0.21)	0.156	−0.15 (0.22)	0.502	0.62 (0.23)	0.008
Multivariate^b^						
Baseline bilirubin	0.62 (0.18)	0.001	0.41 (0.24)	0.083	0.75 (0.27)	0.005
Clustered bilirubin	0.29 (0.15)	0.064	−0.08 (0.21)	0.704	0.45 (0.14)	0.001

^a^ ‘baseline bilirubin’ is the results of regression analysis for clinical outcomes according to the baseline bilirubin levels. ‘Clustured analysis’ is the results of regression analysis for clinical outcomes and repeated measured serum bilirubin levels

^b^ adjusted with age, sex, body mass index, smoking (current and former), baseline FEV_1_ GOLD Global Initiative for Chronic Obstructive Lung Disease., SE standard error

**Table 5:** Regression analysis for the relationship between serum bilirubin levels and mortality^a^

	Whole group		GOLD I-II		GOLD III-IV	
	HR (95% CI)	*P*-value	HR (95% CI)	*P*-value	HR (95% CI)	*P*-value
Univariate						
Baseline bilirubin	1.31 (0.53–3.24)	0.563	0.74 (0.14–3.79)	0.715	1.50 (0.49–4.57)	0.473
Clustered analysis	0.16 (0.02–1.10)	0.062	0.14 (0.01–2.60)	0.188	0.17 (0.01–2.27)	0.180
Multiple^b^						
Baseline bilirubin	1.60 (0.65–3.97)	0.311	1.23 (0.24–6.26)	0.805	1.36 (0.47–3.88)	0.569
Clustered analysis	0.19 (0.03–1.19)	0.076	0.21 (0.01–4.42)	0.315	0.33 (0.03–3.86)	0.377

^a^ ‘baseline bilirubin’ is the results of regression analysis for clinical outcomes according to the baseline bilirubin levels. ‘Clustured analysis’ is the results of regression analysis for clinical outcomes and repeated measured serum bilirubin levels

^b^ Adjusted for age, sex, body mass index, smoking (current and former), and baseline forced expiratory volume in 1 s

*GOLD* Global Initiative for Chronic Obstructive Lung Disease, *HR* hazard ratio, *CI* confidence interval

### Mortality

Serum bilirubin level was not associated with mortality in the whole group or in the subgroups in the analysis of the baseline level or in the clustered analysis (Table 5).

## Discussion

This study investigated the association of serum bilirubin levels with several clinical aspects, such as lung function, exercise capacity, QoL, exacerbation, and mortality, through the Korean COPD cohort study. We found that higher serum bilirubin level was associated with longer 6 MW distance, better QoL, and higher risk of acute exacerbation after adjusting for age, sex, BMI, smoking status, and baseline FEV_1_. When stratifying patients according to severity of airflow limitation, the association with better exercise capacity and better QoL persisted only in the GOLD I-II group, whereas the association with a higher risk of exacerbation persisted only in the GOLD III-IV group.

Bilirubin is a potential antioxidant against peroxyl radicals and protects cells from toxic levels of hydrogen peroxide [3]. Powerful antioxidant actions of bilirubin arise from rapid regeneration to bilirubin via biliverdin reductase after being oxidized back to biliverdin [11, 12]. Additionally, bilirubin attenuates vascular endothelial activation and dysfunction in response to proinflammatory stress [13].

These may explain how higher serum bilirubin levels are associated with better exercise capacity in COPD. This is the first study to demonstrate a positive association between serum bilirubin level and 6 MW distance in COPD patients. However, the association was only observed in the GOLD I-II group. In COPD patients with severe airflow limitation, other factors such as BMI, muscle weight, baseline saturation, level of dyspnea, and lung function have greater influence on exercise capacity. Handgrip strength was independently and positively related to serum total bilirubin level in both sexes among Japanese community-dwelling persons [14]. Reactive oxygen species and reactive nitrogen species are generated in skeletal muscle both during rest and contractile activity [15]. Intense and prolonged exercise can result in oxidative damage to both proteins and lipids in contracting myocytes. Low and physiological levels of reactive oxygen species are required for normal force production in skeletal muscle, but high levels of reactive oxygen species promote contractile dysfunction, resulting in muscle weakness and fatigue [16]. Bilirubin is one of the numerous nonenzymatic antioxidants located within skeletal muscle fibers, and it inhibits both lipid and protein oxidation [14]. In addition, bilirubin attenuates vascular endothelial activation and dysfunction in response to proinflammatory stress [13]. Albumin-bound bilirubin protects human ventricular myocytes against oxyradical damage [17].

However, the relationship of bilirubin and clinical outcomes should be cautiously assessed in various settings when other health statuses could confound the results. Brown et al. showed that higher bilirubin level was associated with lower risk of acute exacerbation of COPD from the secondary analyses of data in the Simvastatin for Prevention of Exacerbations in Moderate-to-Severe COPD (STATCOPE) and the Azithromycin for Prevention of Exacerbations of COPD (MACRO) studies [8]. However, we found contradicting results, as a higher bilirubin level was associated with higher risk of exacerbation of COPD, especially in the GOLD III-IV group. This result could be cautiously explained by the relationship between bilirubin and right-heart function. Poelzl et al. showed that the median total bilirubin level increased with every New York Heart Association class [18]. Moreover, Samsky et al. reported that several echocardiographic indices of right-heart dysfunction were related to elevated total bilirubin levels, with increased portal vein pulsatility index having the best predictive value in patients with exacerbation of chronic heart failure. Abnormal liver function test results were observed in patients with heart failure as a result of impaired perfusion or increased right-sided cardiac pressures [19]. COPD patients with right-sided heart failure were at higher risk of severe exacerbations [20]. Relative pulmonary artery enlargement (ratio of the main pulmonary artery diameter to the aortic diameter > 1) was a significant biomarker for predicting future exacerbation in the COPD gene study [21]. The ratio of the main pulmonary artery diameter to the aortic diameter was positively related with right ventricular pressure, and a high ratio was a significant risk factor of COPD exacerbation in Korean COPD patients [22]. In this study, the relationship between serum bilirubin and exacerbation was only significant in the GOLD III-IV group, which had more severe airflow limitation, but the cardiac function status in this group is not exactly known. In STATCOPE and MACRO, COPD patients with predicted FEV_1_ < 80% were enrolled, and one-third were categorized into the GOLD II group [23, 24]. Moreover, the study duration of STATCOPE (median = 1.74 years) and MACRO (median = 0.55 years) was shorter than that of our study (mean = 5.4 years).

In contrast to the findings of several previous studies, serum bilirubin level was not associated with pulmonary function in COPD patients. Curjuric et al. studied the association of bilirubin with lung function in the Swiss study on Air Pollution and Lung Disease in adults (SAPALDIA) cohort. High bilirubin levels were significantly associated with higher FEV_1_/FVC and forced expiratory flow at 25–75% of the pulmonary volume (FEF_25–75%_) overal l[6]. Leem et al. found significant associations of serum bilirubin levels with FEV_1_, FVC, and FEF_25–75%_ in the general population, especially in never-smokers. Moreover, serum bilirubin levels were related to an annual decline in FEV_1_, FVC, and the FEV_1_/FVC ratio [9]. Apperley et al. reported that bilirubin is inversely related to COPD disease severity and progression. Higher serum bilirubin concentration was associated with a higher FEV_1_ and less annual decline in FEV_1_ [7]. In the study by Apperley et al., participants were active smokers with mild to moderate airflow limitation, defined as FEV_1_ between 55 and 90% predicted, and their mean FEV_1_ was 75% predicted. In the GOLD I-II group of our study, the mean FEV_1_ was 66.7% predicted, and half of the group had FEV_1_ < 70% predicted (data not shown), meaning that airflow limitation was more severe in our study than that in the study by Apperley et al. Milevoj Kopcinovic et al. assessed the association of bilirubin as an oxidative stress marker with stable COPD patients. Although the number of participants was small, the total bilirubin levels were not different between patients with different COPD severities [25].

The association of serum bilirubin level with mortality is controversial, depending on the subtypes of mortality. Its association with overall mortality in COPD has not been reported previously in accordance with our study. In mild COPD, bilirubin was only inversely correlated with coronary heart disease mortality, but not with overall mortality [7].

Our study had several limitations. First, methodologically, the causality between serum bilirubin level and clinical consequences of COPD could not be established. Although it is currently impossible to conduct a prospective study with supplementation of bilirubin to investigate the clinical effects of bilirubin in patients with COPD, pretreatment with gavage of indirect bilirubin attenuated smoking-induced pulmonary injury by suppressing inflammatory cell recruitment and pro-inflammatory cytokine secretion and increasing anti-inflammatory cytokine levels and antioxidant superoxide dismutase activity in a rat model of smoking-induced emphysema [26]. Second, a smaller number of patients were included in our study compared with previous large-cohort studies. However, the strength of our study is that data from repeated measurements over a long-term period were used. We evaluated the association of serum bilirubin levels with clinical outcomes using two different methods: (1) analysis of the relationship between the baseline serum bilirubin level with serial data of various clinical parameters, (2) analysis of all repeated serum bilirubin levels with serial measurements of clinical parameters using the clustered analysis method. Third, this cohort study did not include other factors that may affect bilirubin levels, such as previous medication, alcohol consumption, or diet. Moreover, parameters of cardiac functions were not fully evaluated in KOLD cohort. Therefore, our assumption of relationship between heart failure and high serum bilirubin levels in severe airflow limitation cases could not be proved in this study. Additionally, mortality could not be solely by the effects of acute exacerbation because death was defined as all cause death in our study.

## Conclusion

This is the first COPD cohort study to investigate the relationship of repeatedly measured serum bilirubin levels and COPD-related clinical outcomes every year. A higher serum bilirubin level was independently associated with increased exercise capacity, a better QoL in patients with mild-to-moderate COPD, and a higher risk of exacerbation in those with severe-to-very severe COPD. Bilirubin possesses potential antioxidant and anti-inflammatory effects. However, bilirubin should be cautiously considered as a biomarker for predicting clinical consequences in various settings of COPD.

## Supplementary information

**Additional file 1:.** Serum bilirubin levels over the follow-up period. Serum bilirubin levels did not vary between the visits over the follow-up period.

## Data Availability

The datasets used and/or analysed during the current study are available from the corresponding author on reasonable request. Deidentified participant data may be requested with request form containing reasonable purpose and study protocols to Sang-Do Lee (sdlee@amc.seoul.kr). Permission for use of data would be discussed in the meeting of KOLD Study Group.

## Abbreviations

6 MW: 6-min walking distance
BMI: Body mass index
CAT: COPD Assessment Test
CI: Confidence interval
COPD: Chronic obstructive pulmonary disease
FEV_1_: Forced expiratory volume in 1 s
FVC: Forced vital capacity
GEE: Generalized estimating equations
GOLD: Global Initiative for Chronic Obstructive Lung Disease
KOLD: Korean Obstructive Lung Disease
MACRO: Azithromycin for Prevention of Exacerbations of COPD
OR: Odds ratio
QoL: Quality of life
SAPALDIA: Swiss study on Air Pollution and Lung Disease in Adults
SD: Standard deviation
SGRQ: St. George’s Respiratory Questionnaire
STATCOPE: Simvastatin for Prevention of Exacerbations in Moderate-to-Severe COPD
