# Supplementary material for: Serum bilirubin level is associated with exercise capacity and quality of life in chronic obstructive pulmonary disease
Source: Respir Res. 2019 Dec 9;20:279. doi: 10.1186/s12931-019-1241-5 (PMC6902503; doi:10.1186/s12931-019-1241-5)
Supplement: Supplementary file 1 — Additional file 1:. Serum bilirubin levels over the follow-up period. Serum bilirubin levels did not vary between the visits over the follow-up period. [file 12931_2019_1241_MOESM1_ESM.pdf]

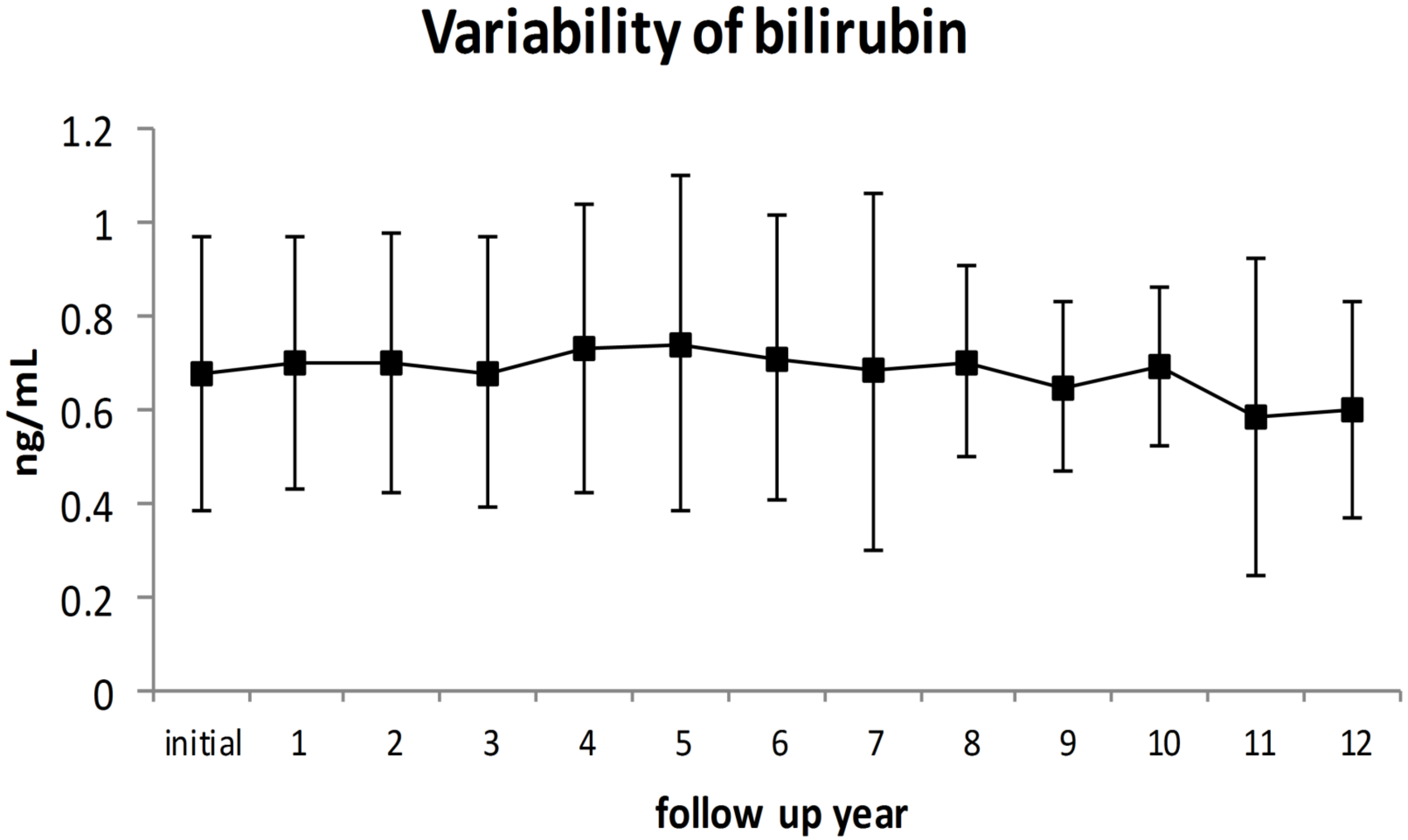

Additional file 1. Serum bilirubin levels over the follow-up period

Serum bilirubin levels did not vary between the visits over the follow-up period.
